# Performance evaluation of the TE Air wireless handheld ultrasound in cardiac applications: a prospective comparative study

**DOI:** 10.1186/s13089-025-00454-0

**Published:** 2025-10-29

**Authors:** Beibei Ge, Mingxia Li, Hanlin Cheng, Zhanru Qi, Xiaoxian Wang, Fen Chen, Zhongqing Shi, Guanjun Guo, Chunjie Shan, Jinyang Qi, Shouhua Luo, Fang Xu, Jing Yao

**Affiliations:** 1 Department of Ultrasound Medicine, Nanjing Drum Tower Hospital, Affiliated Hospital of Medical School, Nanjing University, Nanjing, 210008 People’s Republic of China; 2 School of Biological Sciences and Medical Engineering, Southeast University, Nanjing, 210096 People’s Republic of China; 3 Department of Ultrasound Medicine, Yancheng First People’s Hospital Affiliated of Nanjing University Medical College, Nanjing University, Yancheng, 224000 People’s Republic of China; 4 Medical Imaging Center, Nanjing Drum Tower Hospital, Affiliated Hospital of Medical School, Nanjing University, Nanjing, 210008 People’s Republic of China

**Keywords:** Handheld ultrasound, Wireless, TE Air, Cardiac ultrasound

## Abstract

**Aim:**

To evaluate the reliability and reproducibility of the TE Air wireless handheld ultrasound device in clinical cardiac imaging by comparing its performance with a high-end reference system.

**Methods:**

161 patients for good-quality echocardiographic images were included in this prospective study. Each patient underwent sequential imaging using both the TE Air device (Mindray) and the high-end reference device (Philips EPIQ 7 C). Nine standard cardiac views were acquired. Image quality was assessed manually by two blinded echocardiographers and via proprietary AI software, respectively. The following key parameters were analyzed basing on the images: diastolic thickness of interventricular septal (IVSTd) and left ventricular posterior wall (LVPWTd), left ventricular end-diastolic (LVDd) and end-systolic diameter (LVDs), aortic diameter (AOD), left atrial anteroposterior diameter (LAD), Early (E) and late (A) diastolic velocities of the mitral valve in PW mode, as well as early diastolic velocities at the septal (EmS) and lateral (EmL) mitral annulus. Regional wall motion abnormality (RWMA), bicuspid aortic valve (BAV), atrial septal defect (ASD), left ventricular ejection fraction (LVEF) and valvular regurgitation degree were independently evaluated.

**Results:**

The TE Air demonstrated comparable image quality to the high-end reference system in both manual (64.95 ± 1.24 vs. 64.19 ± 1.63, *P* = 0.28) and AI-based evaluations (65.07 ± 1.02 vs. 63.80 ± 1.68, *P* = 0.06). Structural measurements showed high inter-device consistency, with ICCs of 0.77/0.74 for IVSTd/LVPWTd, 0.95/0.96 for LVDd/LVDs, and 0.82/0.98 for AOD/LAD (all *P* < 0.001). Functional parameters also demonstrated strong agreement (ICC: 0.91/0.92 for mitral E/A waves; 0.79/0.85 for EmS/EmL; *P* < 0.001). The TE Air had sensitivities of 81.8% for RWMA, 100% for ASD and BAV, and 93.5% for LVEF < 50%. Diagnostic agreement was excellent for LVEF (κ = 0.96, *P* < 0.001) and valvular regurgitation (weighted κ = 0.89, *P* < 0.001).

**Conclusion:**

The TE Air wireless handheld ultrasound device exhibits high agreement with high-end reference device in image quality, measurements, and clinical diagnoses, supporting its potential for widespread use in point-of-care ultrasound (POCUS) clinical applications.

**Graphical Abstract:**

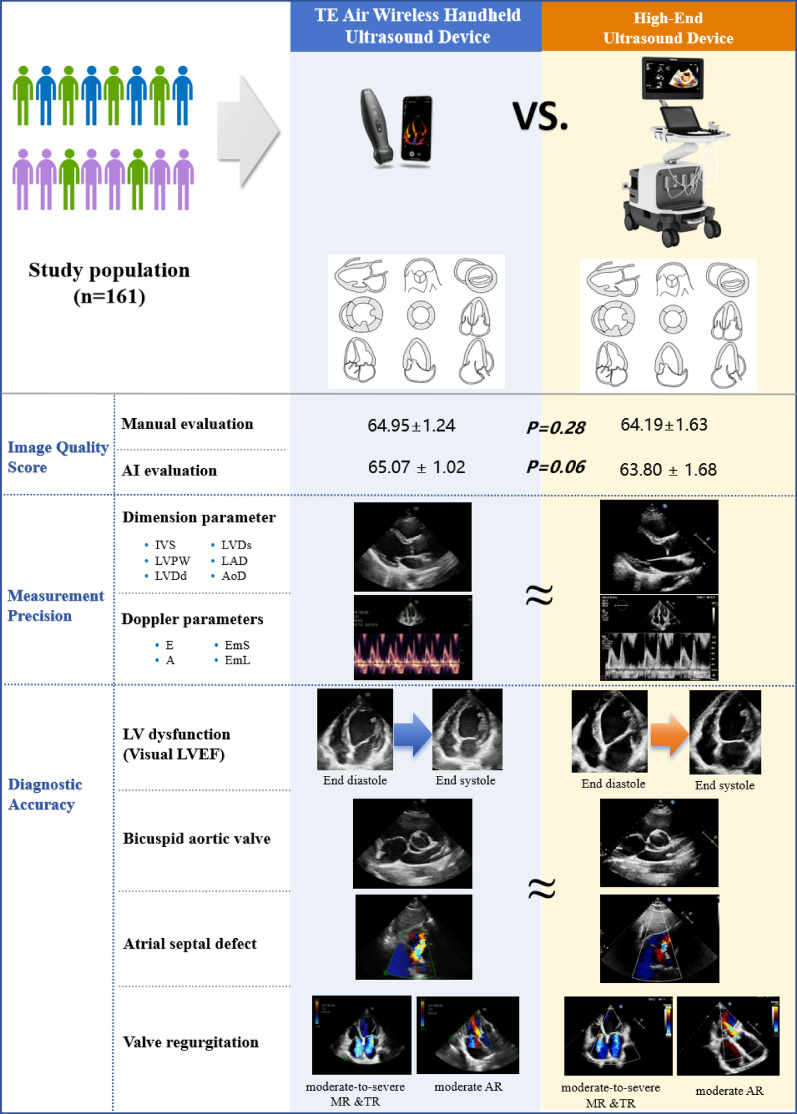

**Supplementary Information:**

The online version contains supplementary material available at 10.1186/s13089-025-00454-0.

## Background

Transthoracic echocardiography (TTE) is the most widely used non-invasive imaging modality for assessing cardiac structure and function, owing to its accessibility, cost-effectiveness, and safety. Despite its clinical utility, traditional TTE systems are often bulky and lack portability, limiting their use in emergency and critical care settings. To address these limitations, point-of-care ultrasound (POCUS) has emerged as a valuable tool, enabling rapid bedside assessments that support timely diagnosis and therapeutic decision-making [1, 2].

Traditional POCUS systems, while portable, are typically cart-based and still impose logistical challenges in confined or high-demand environments [2–4]. Recent advancements in ultrasound technology have led to the development of handheld devices, which offer unprecedented portability and ease of use [5, 6]. While handheld ultrasound devices mark a major advancement in portable diagnostics, their miniaturization may raise concerns about compromised image quality and diagnostic accuracy, particularly in demanding applications like cardiac imaging. Multiple studies have shown that handheld ultrasound devices achieve diagnostic accuracy comparable to cart-based systems for common conditions and procedures [7–9]. Several authors have evaluated the role of portable ultrasound devices in intensive medicine and in pre-hospital emergency medicine [10, 11]. These devices have demonstrated promising performance in various clinical applications, including bedside procedures, musculoskeletal imaging, abdominal pathology assessment, and evaluation of left ventricular function [8, 12–14].

**Fig. 1:**
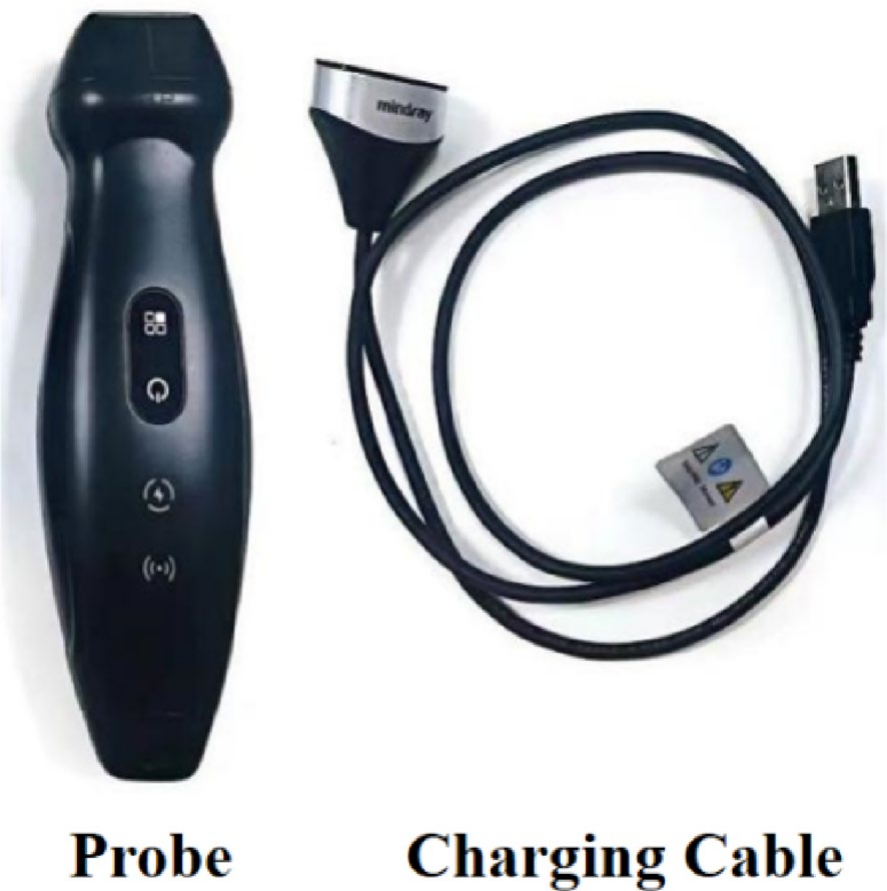
TE Air wireless handheld ultrasound devices

The TE Air is a novel wireless handheld ultrasound device developed by Mindray (Fig. 1). Initial studies indicate that the TE Air achieves sufficient image clarity, color Doppler flow imaging (CDFI) performance, and operational simplicity for clinical applications, allowing physicians to make accurate diagnoses despite its compact design [15]. A recent cross-sectional study evaluated six portable ultrasound devices, assessing their overall image quality, ease of use, and user satisfaction while providing a comparative ranking. For the apical 4-chamber view, key evaluation criteria included endocardial definition, valve leaflet clarity, lateral tricuspid valve annulus visibility, far-field resolution, and color flow Doppler performance in the left ventricular outflow tract and mitral valve. Among the handheld devices tested, Mindray TE Air received the highest rating for this view [16]. Mindray TE Air was one of the top 3 highest-rated handhelds for overall satisfaction with ease of use and image quality [16]. However, the TE Air has yet to be systematically evaluated for cardiac imaging. This study, therefore, aims to assess the reliability and reproducibility of the TE Air wireless handheld ultrasound device in cardiac ultrasound applications.

## Methods

### Study population

This study recruited consecutive patients who had been referred to the echocardiography laboratories of Nanjing Drum Tower Hospital and Yancheng First People’s Hospital for routine clinical cardiac assessment. Patients that had good echocardiographic imaging windows were included. Patients were excluded if they had poor-quality echocardiographic images. Written informed consent was obtained from all subjects prior to inclusion, and the protocol of this study was approved by the Nanjing Gulou Hospital ethics committee.

### Cardiac ultrasound examinations

Cardiac ultrasound examinations were conducted by two experienced cardiologist (more than 6500 examinations/year, more than 5 years of experience in cardiac ultrasound), with all images stored in Digital Imaging and Communication in Medicine (DICOM) format following manufacturer guidelines. Each participant underwent sequential imaging using both the TE Air wireless handheld ultrasound device (1.8–4.5 MHz, Mindray, Shenzhen, China) and a conventional high-end ultrasound system (EPIQ 7 C with S5-1 or X5-1 transducers, 1.0–5.0 MHz, Philips, Andover, MA, USA). Patients were instructed to rest for 5 min before imaging and were positioned in the left lateral decubitus position to optimize image acquisition. To minimize respiratory interference, images were captured at end-expiration whenever possible.

The following echocardiographic views were acquired: (1) parasternal long axis view of left ventricle (PLAX); (2) parasternal short axis view of aortic valve (PSAXGV); (3) parasternal short axis view of left ventricle at mitral valve level (PSAXMV); (4) parasternal short axis view of left ventricle at papillary muscle level (PSAXPM); (5) parasternal short axis view of left ventricle at apical level (PSAXA); (6) apical four chamber view (A4C); (7) apical five chamber view (A5C); (8) apical two chamber view (A2C); (9) apical three chamber view (A3C). In addition, pulse wave (PW) Doppler imaging of mitral valve inflow and PW tissue Doppler imaging (TDI) at the septal and lateral mitral valve annulus were performed in the A4C view. These measurements provided comprehensive functional and structural assessments of cardiac performance.

### Accuracy assessment of cardiac imaging

A comprehensive evaluation of the TE Air wireless handheld ultrasound device for cardiac imaging was conducted, focusing on three key aspects: image quality, measurement reliability, and diagnostic accuracy. The inter-observer reproducibility of echocardiographic parameters (including image quality score, dimension and doppler parameters) was assessed in a random subset of 40 participants.

### Image quality assessment

Image quality for all standard echocardiographic views was evaluated by two experienced echocardiographers (more than 6500 examinations/year, more than 5 years of experience in cardiac ultrasound) and an artificial intelligence (AI) software developed by our team. The echocardiographers were blinded to each other’s assessments and patient details to ensure objectivity. A detailed scoring system was used to assess image quality based on gain requirements and the visualization of major and minor cardiac structures across the nine standard views. The cumulative score for each patient was derived from the evaluation of these views.The scoring criteria for image quality are outlined in Supplemental Table 1 [17].

### Measurement reliability

Cardiac parameters obtained from both the TE Air and the high-end reference system were measured offline by the Radiant DICOMViewer software by two experienced echocardiographers. The following parameters were assessed in B-mode: Interventricular septal thickness in diastole (IVSTd), left ventricular posterior wall thickness in diastole (LVPWTd), left ventricular end-diastolic diameter (LVDd), left ventricular end-systolic diameter (LVDs), aortic diameter (AOD) referring to the dimension at the sinotubular junction, and left atrial anteroposterior diameter (LAD). In the parasternal long-axis view, IVSTd, LVPWTd, LVDd, and AOD are measured at end-diastole, while LAD was measured at end-systolic. Additionally, in pulsed Doppler (PW) mode, the early (E wave) and late (A wave) diastolic mitral inflow velocities were recorded. In tissue Doppler imaging (TDI) mode, the early diastolic mitral annular velocities were measured at both the septal (EmS) and lateral (EmL) walls. The parameters were measured according to the JASE guideline [17]. All echocardiographers performing the measurements were blinded to patient information to eliminate bias.

### Diagnostic accuracy

Regional wall motion abnormality (RWMA), bicuspid aortic valve (BAV), atrial septal defect (ASD), left ventricular ejection fraction (LVEF) < 50% based on visual estimation and qualitative moderate or greater regurgitation of the mitral, tricuspid, or aortic valves were independently evaluated.

### Statistical analysis

The normality of variables were assessed using the Kolmogorov-Smirnov test and P-P plots. Continuous variables were presented as mean ± standard deviation, and categorical variables are presented as frequencies and percentages. Comparison between cardiac ultrasound image quality scores were performed using paired t-test. The agreement in image quality scores and echocardiographic measurements between the two devices was assessed using Bland-Altman plots and the Intraclass Correlation Coefficient (ICC). Consistency between the diagnostic results was analyzed using Cohen’s Kappa test. All statistical tests were two-tailed, and a *P* values < 0.05 was considered statistically significant. Data analysis was performed using commercially available statistical software (SPSS version 27.0; SPSS, Inc, Chicago, IL).

## Results

A total of 161 patients were enrolled (82 females, 51%). The mean age was 53.94 ± 16.48 years, with an average body mass index (BMI) of 23.61 ± 2.82 kg/m². The study population included individuals with multiple heart diseases, ensuring a diverse representation of clinical scenarios for device evaluation. Table 1 summarizes the cardiovascular diseases diagnosed using the high-end ultrasound device, with some patients presenting multiple conditions (Table 1).

**Table 1:** The cardiovascular diseases diagnosed by high-end ultrasound device

Cardiovascular diseases	Number
No structural heart disease	83
Regional wall motion abnormality	11
Hypertrophic cardiomyopathy	2
Bicuspid aortic valve	8
Atrial septal defect	5
Left ventricular Apical Thrombus	3
Pericardial effusion	8
Valve stenosis (mitral, tricuspid, or aortic)	7
Moderate to severe valvular regurgitation (mitral, tricuspid, or aortic)	46

### Echocardiographic image quality score

The average manual image quality score for the high-end reference device was 64.19 ± 1.63, while the TE Air achieved a score of 64.95 ± 1.24. AI-based evaluation yielded a score of 65.07 ± 1.02 for the TE Air, compared to 63.80 ± 1.68 for the high-end device. No statistically significant differences were observed between the two devices in either manual (*P* = 0.28) or AI-based (*P* = 0.06) assessments. Bland-Altman plots (Supplemental Fig. 1) confirmed the absence of value-dependent bias between the devices. The inter-observer reproducibility of image quality score for both devices, as well as the reproducibility of the AI’s twice score, were both good. Supplemental Table 2 demonstrates the inter-observer reproducibility.

### Evaluation of echocardiographic measurements

The IVSTd and LVPWTd showed moderate correlation coefficients of 0.77 and 0.74, respectively, between the two devices. The ICCs for LVDd and LVDs were 0.95 and 0.96, respectively, indicating excellent agreement. Similarly, the AOD and LAD values demonstrated strong consistency, with correlation coefficients of 0.82 and 0.98, respectively.

**Fig. 2:**
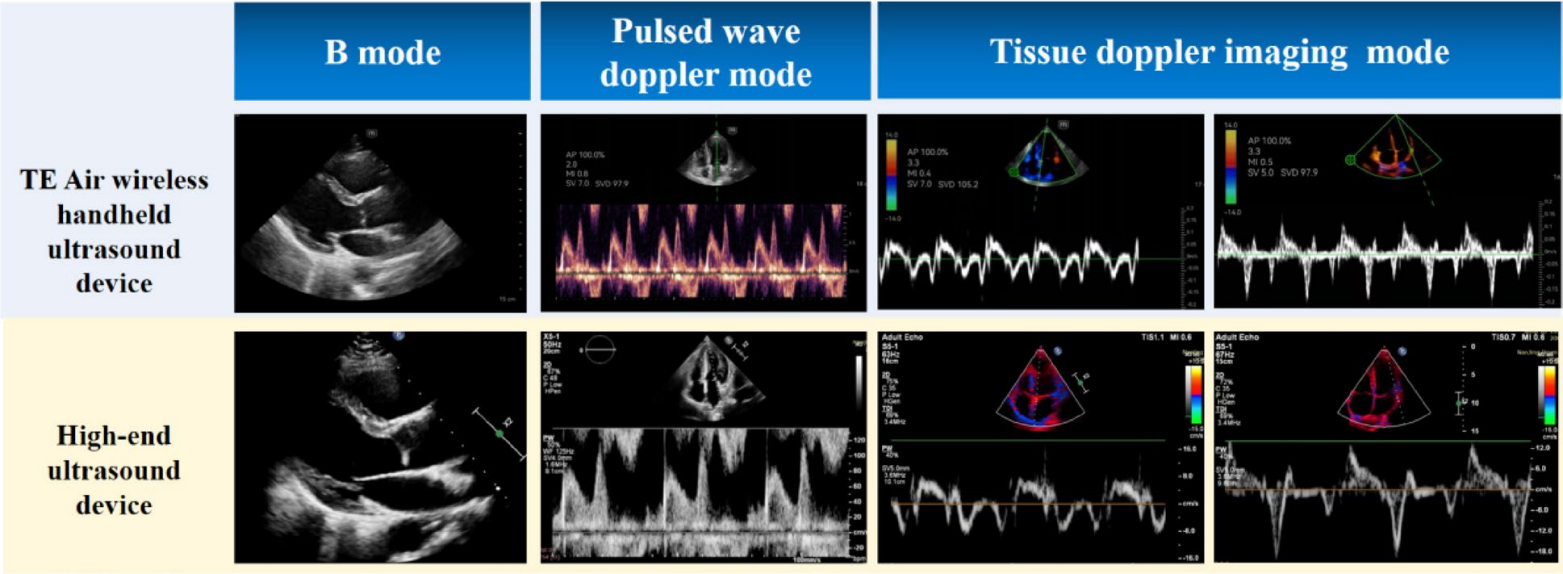
Representative views of patients for B, pulsed wave doppler and tissue doppler imaging

For the Doppler parameters, the ICCs for the E and A velocities were 0.91 and 0.92, respectively. The ICCs for EmS and EmL were 0.79 and 0.85, respectively, indicating good agreement between the two devices. Table 2 provides detailed information on the dimension and Doppler parameters, along with their corresponding ICC values. Representative patient views for B-mode, PW mode, and TDI mode are presented in Fig. 2. Bland-Altman plots further confirmed the absence of value-dependent bias between the devices. Good inter-observer reproducibility was observed for all measurements (Supplemental Table 2).

**Table 2:** Left ventricle dimension and doppler measurement parameters

Parameters		High-end ultrasound device	TE Air ultrasound device	ICC	95%CI
Dimension parameters	IVSTd(cm)	0.92 ± 0.11	0.91 ± 0.14	0.77*	(0.68, 0.84)
	LVPWTd(cm)	0.87 ± 0.10	0.88 ± 0.13	0.74*	(0.64, 0.82)
	LVDd(cm)	4.82 ± 0.63	4.78 ± 0.61	0.95*	(0.93, 0.97)
	LVDs(cm)	3.12 ± 0.84	3.01 ± 0.81	0.96*	(0.94, 0.97)
	AoD(cm)	2.78 ± 0.31	2.77 ± 0.34	0.82*	(0.74, 0.87)
	LAD(cm)	3.44 ± 0.65	3.41 ± 0.66	0.98*	(0.97, 0.99)
Doppler parameters	E(cm/s)	75.90 ± 16.65	74.29 ± 15.60	0.91*	(0.87, 0.94)
	A(cm/s)	74.16 ± 19.57	70.99 ± 18.96	0.92*	(0.88, 0.95)
	EmS (cm/s)	7.96 ± 2.16	7.94 ± 2.23	0.79*	(0.70, 0.86)
	EmL (cm/s)	10.57 ± 3.13	11.44 ± 3.63	0.85*	(0.78,0.90)

Values are presented as mean ± SD

ICC: Intraclass Correlation Coefficient; CI: confidence interval; IVSTd: Interventricular septal thickness in diastole; LVPWTd: left ventricular posterior wall thickness in diastole; LVDd: left ventricular end-diastolic diameter༛LVDs: left ventricular end-systolic diameter༛AoD: aortic diameter༛ LAD: left atrial anteroposterior diameter༛ E: early diastolic mitral valve inflow velocity༛A: late diastolic mitral valve inflow velocity༛EmS: septal early diastolic mitral annular velocity༛EmL: lateral early diastolic mitral annular velocity

*P < 0.001

### Evaluation of diagnostic result

**Fig. 3:**
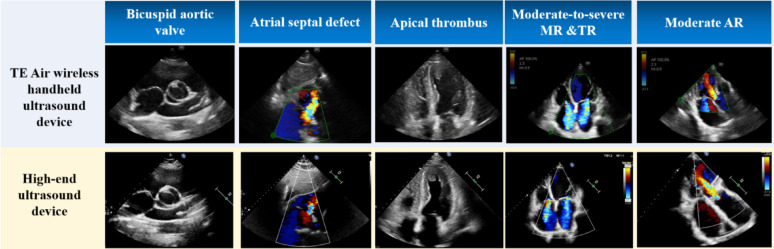
Diagram of different cardiovascular diseases in TE Air and high-end ultrasound devices

Among the 161 participants, High-end ultrasound diagnosis confirmed 11 cases with regional wall motion abnormality (RWMA), 8 cases of bicuspid aortic valve (BAV), and 5 cases of atrial septal defect (ASD). The TE Air wireless handheld ultrasound detected 9 cases with RWMA, 8 cases of BAV, and 5 cases of ASD. Both devices identified left ventricular apical thrombosis in 3 patients (Fig. 3). Table 3 shows the sensitivity and specificity between the two devices.

Left ventricular ejection fraction (LVEF) was visually estimated using A4C and A2C views, with results classified as preserved EF ( ≧ 50%) or reduced EF (< 50%) according to the 2021 ESC Heart Failure guidelines [18]. Both devices identified 29 patients with LVEF < 50% and 130 patients with LVEF ≧ 50%. Two patients showed discordant results, with LVEF ≧ 50% on the TE Air but < 50% on the high-end device. The Kappa for agreement between the devices was 0.96 (*P* < 0.001). The sensitivity and specificity of TE Air in diagnosing LVEF < 50% were 93.5% and 100%.

Valvular regurgitation grades were assessed qualitatively based on established guidelines [19, 20], including valve morphology and color flow assessment. Moderate or greater regurgitation was classified as positive, with cases further subdivided into moderate, moderate-severe, or severe. Additionally, some patients exhibited moderate or greater regurgitation in multiple valves. Among 187 recorded regurgitation cases, 164 showed identical grades on both devices. Of the 23 differing cases, 21 (91%) cases were graded higher on TE Air. Despite these differences, the devices exhibited substantial agreement, with a weighted Cohen’s Kappa of 0.89 (95% CI: 0.84–0.93; *P* < 0.001) (Table 4). Figure 3 illustrates representative different cardiovascular diseases in both devices.

**Table 3:** Sensitivity and specificity for the diagnosis of cardiovascular diseases between TE air and high-end ultrasound devices

	Number, *n* (%)	Sensitivity, %	Specificity, %
RWMA	11 (7)	81.8	100
BAV	8 (5)	100	100
ASD	5 (3.1)	100	100
LVEF < 50%	31 (19)	93.5	100

RWMA, regional wall motion abnormality; BAV, bicuspid aortic valve; ASD, atrial septal defect; LVEF, left ventricular ejection fraction

**Table 4:** Diagnosis of valvular regurgitation in the same patient under two ultrasonic devices

	High-end ultrasound device				
	Regurgitation	Negative	Moderate	Moderate-to-severe	Severe
TE Air ultrasound device	Negative	109	2	0	0
	Moderate	9	15	0	0
	Moderate-to-severe	0	4	4	0
	Severe	0	2	6	36

## Discussion

This pilot study demonstrates an advanced wireless handheld ultrasound device that seamlessly integrates with smart mobile platforms, significantly enhancing imaging mobility. In this study, we specifically evaluated the consistency and reproducibility of the TE Air for echocardiography, focusing on image quality, measurement precision, and diagnostic outcomes. Our findings confirm that the TE Air maintains high consistency and reproducibility when compared to High-end ultrasound device. Despite a limited number of examinations and clinical use cases, the results indicate a broad potential for POCUS clinical application.

Accurate assessment of cardiac dimensions, ventricular wall thickness, and valve Doppler parameters relies on optimal image acquisition. Good-quality images are pivotal for precise parameter measurement and accurate diagnosis. In this study, we included only subjects with good-quality echocardiographic images because we believed that this is an important prerequisite for a reliable analysis. By scoring the cardiac structures displayed across multiple standard echocardiographic views and comparing overall scores, we observed no statistically significant differences between the manual and AI-based score for the two devices. Its B-mode image quality are comparable to those of the high-end system. These results indicate that the TE Air effectively visualizes cardiac anatomy, aiding in diagnosis and supporting subsequent clinical management.

The TE Air device supports a comprehensive range of imaging modes, including B-mode, M-mode, color Doppler flow imaging (CDFI), PW Doppler and tissue Doppler imaging (TDI), meeting diverse clinical demands for echocardiography. The TE Air has shown superior liver and kidney vasculature imaging capabilities, surpassing other handheld models in B-mode, color flow, and PW Doppler for diagnostic-grade image quality [15]. Our analysis of B-mode and M-mode imaging focused on measuring cardiac dimensions and wall thickness, revealing strong consistency with high-end systems. For PW Doppler and TDI assessments, we evaluated mitral inflow velocities and mitral annulus velocities, respectively, demonstrating excellent agreement between the TE Air and reference systems.

We evaluated the diagnostic accuracy of TE Air for several cardiovascular conditions, including RWMA, BAV, and LVEF. The results demonstrated sensitivities of 81.8% for RWMA, 100% for BAV, and 93.5% for LVEF < 50%. The LVEF assessment showed strong agreement between the two devices, with a Kappa value of 0.96. Color Doppler flow imaging is crucial for evaluating valve stenosis, regurgitation, and intracardiac shunts. Our study primarily focused on ASD and valve regurgitation. The sensitivity of TE Air in diagnosing ASD was 100%. Valve regurgitation assessments agreed strongly between the two devices, with a weighted Cohen’s kappa of 0.89. However, in practical use, the high-end device has an edge over the TE Air. High-end ultrasound system offers a detailed adjustment of the flow parameters via scale, pulse repetition frequency, wall filter, color gain, and dynamic rang. Compared to high-end devices, the detailed analysis of color flow are inferior. One main limitation is that while TE Air technology can assess color flow, Doppler spectral analysis and continuous adaptation to various velocity ranges is not available. This may result in relatively crude evaluations of conditions such as valvular regurgitation and intracardiac shunts. In particular, the inability to continuously adjust the blood flow velocity range can easily lead to overflow of flow signals, which may subsequently overestimate the severity of valvular regurgitation. Additionally, the TE Air lacks continuous wave (CW) Doppler mode capability, which precludes the measurement of high-velocity blood flow. This limitation restricts its applicability in evaluating conditions associated with high-velocity jets, such as valvular stenosis, prosthetic valve dysfunction, ventricular septal defects and pulmonary artery hypertension. For comprehensive echocardiographic assessments in such cases, high-end systems with CW Doppler functionality remain indispensable.

Although TE Air has the limitations in color flow, it remains a valuable tool for expanding the scope of cardiac assessment, particularly in emergency situations, intensive care unit (ICU) evaluations, and bedside examinations. Additionally, the device’s ability to produce high-quality images facilitates remote consultations, enhancing diagnostic accessibility and convenience.

## Limitation

This study has several limitations that should be acknowledged. First, the diagnostic accuracy of ultrasound is highly operator-dependent, particularly with portable devices where examiner expertise plays an even more critical role. In this study, LVEF, RWMA, and valvular regurgitation were all assessed using visual estimation. It is noteworthy that for LVEF values at the borderline of 50%, mild RWMA, and valvular regurgitation at the interface between two grading levels, the evaluation results may have certain variations due to differences in operator experience. While these methods are widely used in POCUS clinical practice, they are inherently prone to inter-observer variability. Second, this study included only outpatient cardiovascular disease patients and did not include any who were on mechanical ventilation in the ICU. Future studies should include a broader range of cardiovascular disease types and diverse clinical settings to further validate the TE Air’s performance. Third, while the study demonstrated strong agreement between the TE Air and the high-end reference system, the sample size and double-center design may limit the generalizability of the results. Multi-center studies with larger and more diverse patient populations are needed to further validate the device’s performance and reliability in real-world clinical settings. Despite these limitations, the TE Air represents a significant advancement in portable ultrasound technology, offering a practical and reliable tool for point-of-care cardiac imaging in many clinical scenarios. Fourth, we have not compared the TE Air wireless handheld ultrasound with other handheld ultrasound devices. Comparative studies on various handheld ultrasound devices for different cardiovascular diseases are necessary. This will help clarify the advantages of TE Air in diagnosing cardiovascular conditions compared to other handheld ultrasound devices. Additionally, while we were blinded to baseline patient image, the device (TE Air handheld vs. high-end system) could still be identified during evaluation, potentially introducing bias due to inherent differences which is a limitation unavoidable with current technology. Lastly, and of equal importance, in this study, patients with favorable image quality were selected to ensure a robust basis for comparison. While this selection was necessary to evaluate inter-device reliability and reproducibility, it limits the conclusiveness of this study regarding image quality effects. How much image quality impacts TE Air measurement variability is a question for future research.

## Conclusion

The TE Air wireless handheld ultrasound device demonstrates a high level of reliability in assessing cardiac structure and function, offering a viable alternative to high-end equipment for POCUS clinical applications. Its portability, ease of use, and ability to produce diagnostic-grade images make it a promising tool for expanding access to echocardiography in diverse clinical settings.

## Supplementary Information

Below is the link to the electronic supplementary material.

Supplementary Material 1.

## Data Availability

The data used for supporting the findings of this study are available from the corresponding authors upon request.
