# Supplementary material for: Performance evaluation of the TE Air wireless handheld ultrasound in cardiac applications: a prospective comparative study
Source: Ultrasound J. 2025 Oct 29;17:55. doi: 10.1186/s13089-025-00454-0 (PMC12572465; doi:10.1186/s13089-025-00454-0)
Supplement: Supplementary file 1 — Supplementary Material 1. [file 13089_2025_454_MOESM1_ESM.docx]

Supplemental Table 1 The scoring criteria for image quality

| View | Scoring items | Scoring Criteria | Score |
| --- | --- | --- | --- |
| PLAX | Gain | Proper gain and contrast | 1 |
|  | Main Structures | LV, LVOT, IVS, LVPW, LA, RV | 4 |
|  | Minor Structures | AMV and PMV | 1 |
|  |  | Ao | 1 |
| A2C | Gain | Proper gain and contrast | 1 |
|  | Main Structures | LV, LA | 4 |
|  | Minor Structures | Clear MV | 2 |
|  |  | RV | -1 |
| A3C | Gain | Proper gain and contrast | 1 |
|  | Main Structures | LV, LVOT, IVS, LVPW, LA, AO | 5 |
|  | Minor Structures | Clear MV | 1 |
| A4C | Gain | Proper gain and contrast | 1 |
|  | Main Structures | LV, LA, RV, RA, IVS, IAS | 4 |
|  | Minor Structures | Clear AMV and PMV | 1 |
|  |  | Clear TV | 1 |
| A5C view | Gain | Proper gain and contrast | 1 |
|  | Main Structures | LV, RV, IVS, AO | 4 |
|  | Minor Structures | Clear MV | 0.5 |
|  |  | LA | 0.5 |
|  |  | RA | 0.5 |
|  |  | Clear TV | 0.5 |
| PSAX_GV_ | Gain | Proper gain and contrast | 1 |
|  | Main Structures | LA, RVOT, AO | 5 |
|  | Minor Structures | AoV (if there is an echo of the active valve) | 1 |
|  |  | Main pulmonary artery | 1 |
|  |  | RA | 1 |
| PSAX_MV_ | Gain | Proper gain and contrast | 1 |
|  | Main Structures | IVS, MV, LVIW and LVILW | 5 |
|  | Minor Structures | LVAW | 1 |
|  |  | LVLW | 1 |
|  |  | Clear AMV and PMV | 1 |
| PSAX_PM_ | Gain | Proper gain and contrast | 1 |
|  | Main Structures | IVS, PM, LVIW and LVILW | 5 |
|  | Minor Structures | Clear APM and PPM | 1 |
|  |  | LVAW | 1 |
|  |  | LVLW | 1 |
| PSAX_A_ | Gain | Proper gain and contrast | 1 |
|  | Structures | Display complete myocardium at the left ventricular apex | 4 |
|  |  | Display 3/4 of the myocardium at the left ventricular apex | 3 |
|  |  | Display 1/2 of the myocardium at the left ventricular apex | 2 |
|  |  | Display＜1/2 of the myocardium at the left ventricular apex | 0 |
|  |  | RV | -0.5 |

Supplemental Table 2 The inter-observer reproducibility of echocardiographic parameters

| Parameters | | High-end ultrasound device ICC (95% CI) | TE Air ultrasound device  ICC (95% CI) |
| --- | --- | --- | --- |
| Image quality score | Manual | 0.85 (0.73，0.92) | 0.87 (0.76，0.93) |
|  | AI | 0.92 (0.85，0.96) | 0.89 (0.79，0.94) |
| Dimension parameters | IVSTd | 0.90 (0.83，0.95) | 0.89 (0.80，0.94) |
|  | LVPWTd | 0.88 (0.79，0.94) | 0.90 (0.82，0.95) |
|  | LVDd | 0.96 (0.93，0.98) | 0.94 (0.90，0.97) |
|  | LVDs | 0.97 (0.95，0.99) | 0.97 (0.94，0.98) |
|  | AoD | 0.90 (0.83，0.95) | 0.89 (0.80，0.94) |
|  | LAD | 0.97 (0.95，0.99) | 0.96 (0.92，0.98) |
| Doppler parameters | E | 0.90 (0.82，0.95) | 0.86 (0.74，0.92) |
|  | A | 0.93 (0.86，0.96) | 0.92 (0.84，0.96) |
|  | EmS | 0.88 (0.78，0.94) | 0.88 (0.79，0.94) |
|  | EmL | 0.91 (0.83，0.95) | 0.89 (0.79，0.94) |

Supplemental figure 1

| 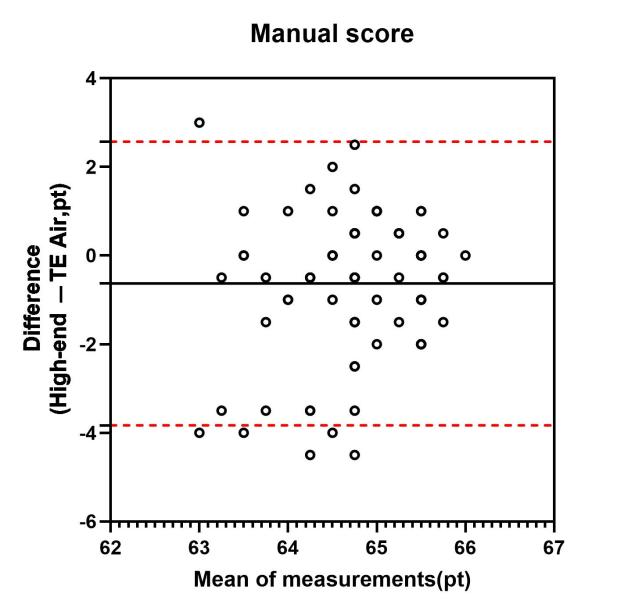 | 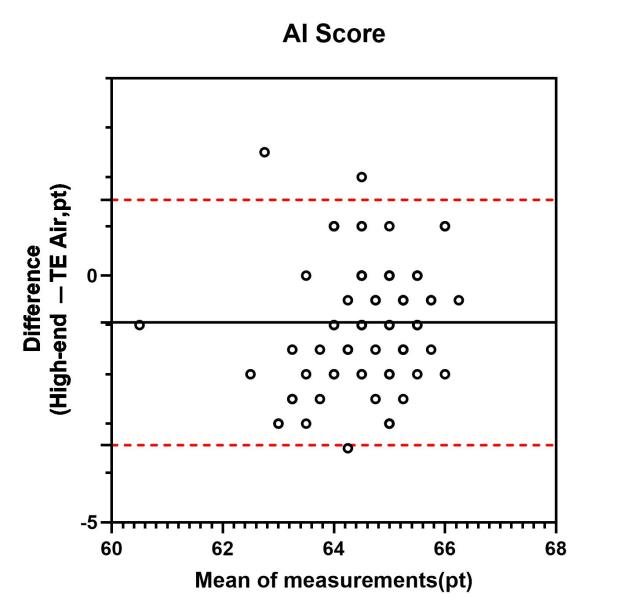 |
| --- | --- |
| 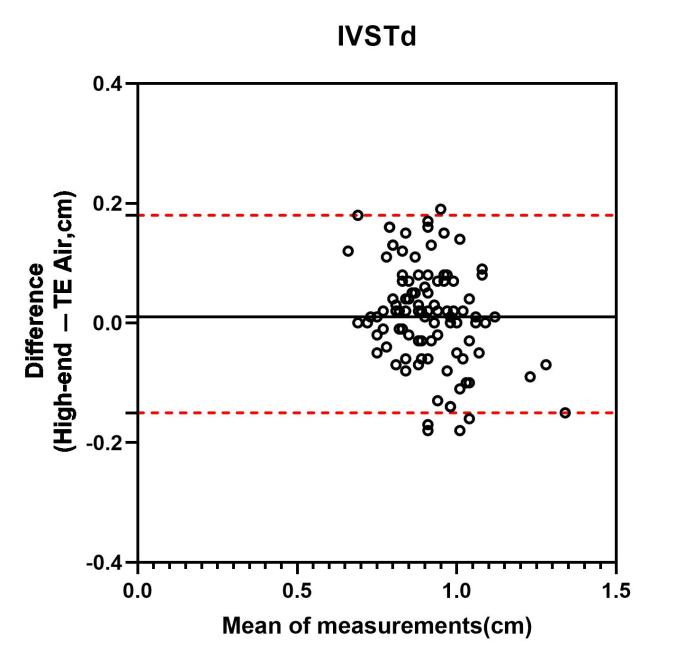 | 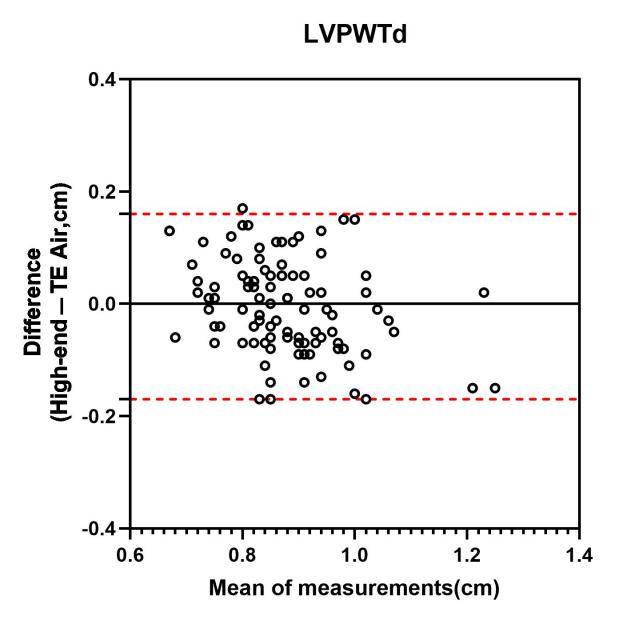 |
| 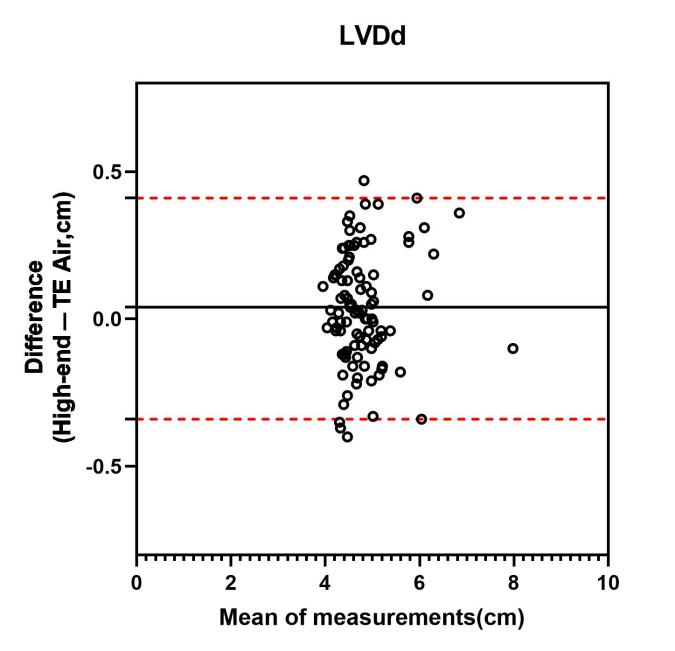 | 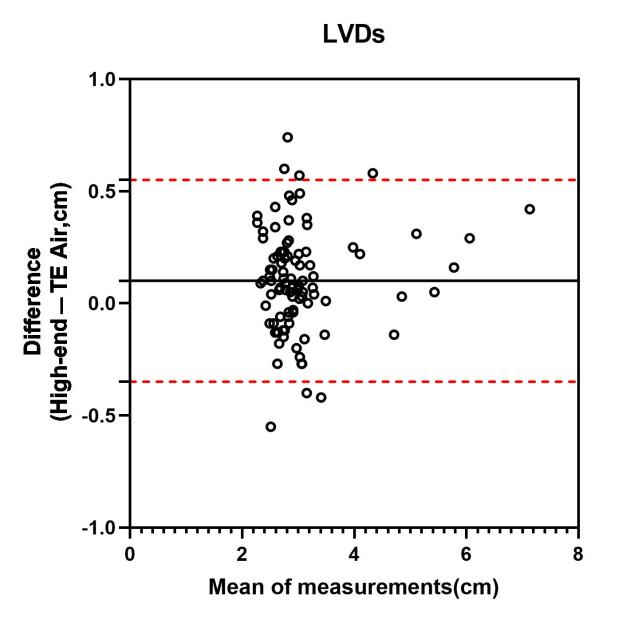 |
| 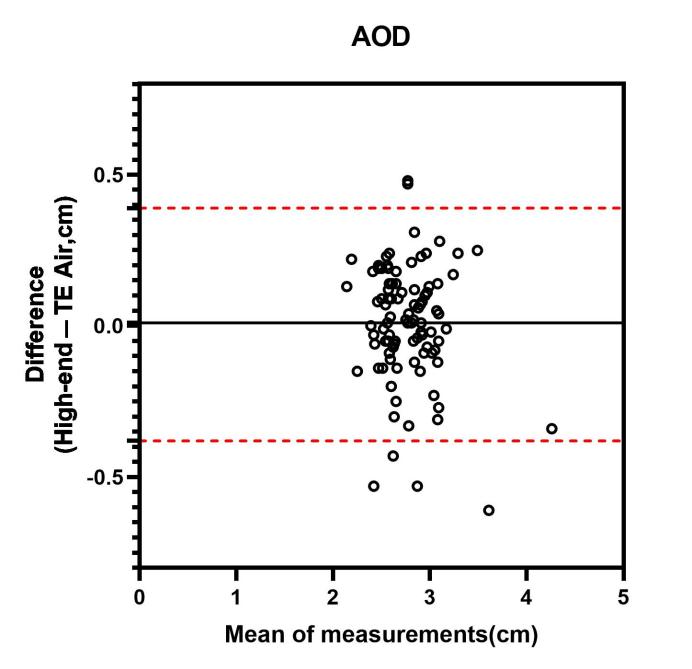 | 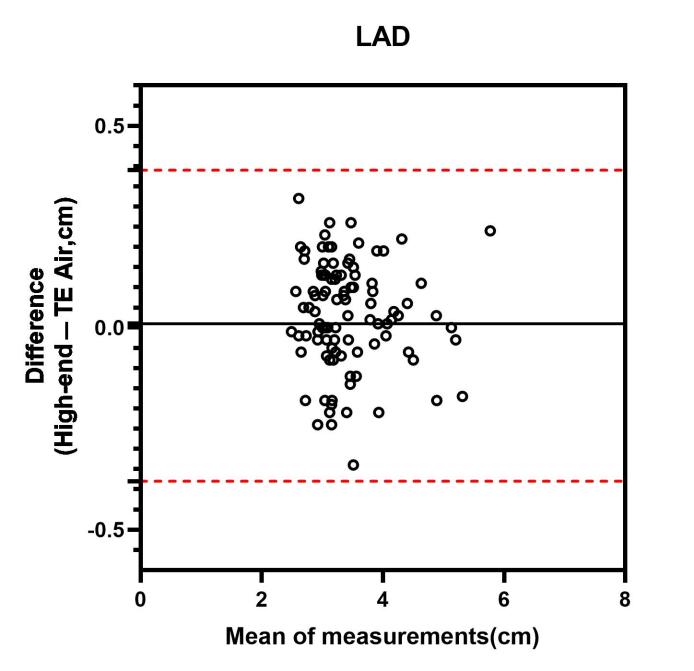 |

| 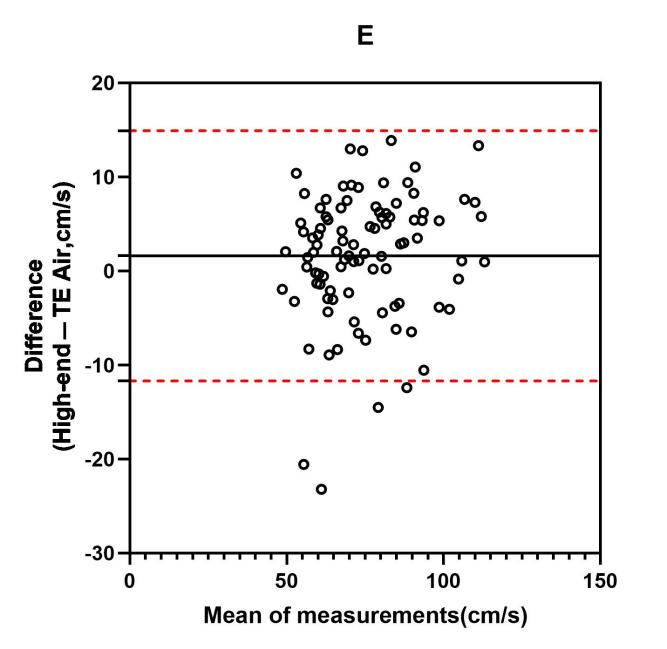 | 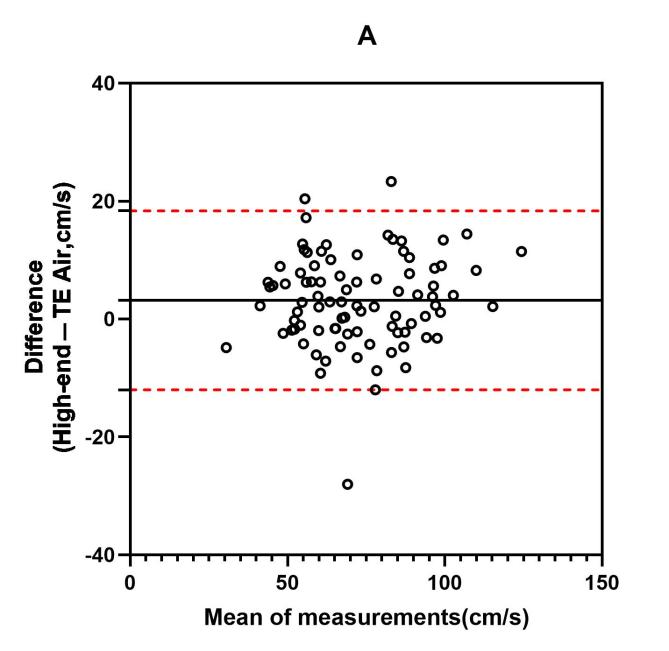 |
| --- | --- |
| 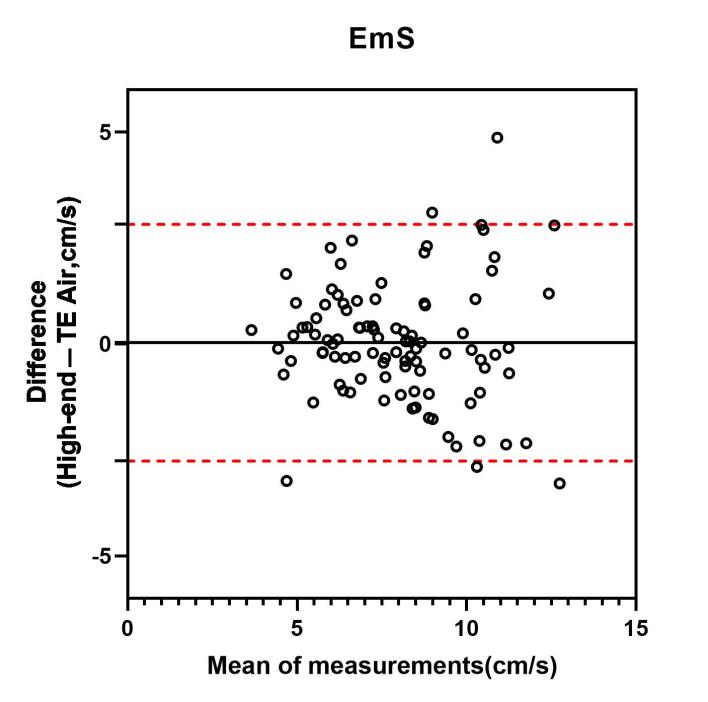 | 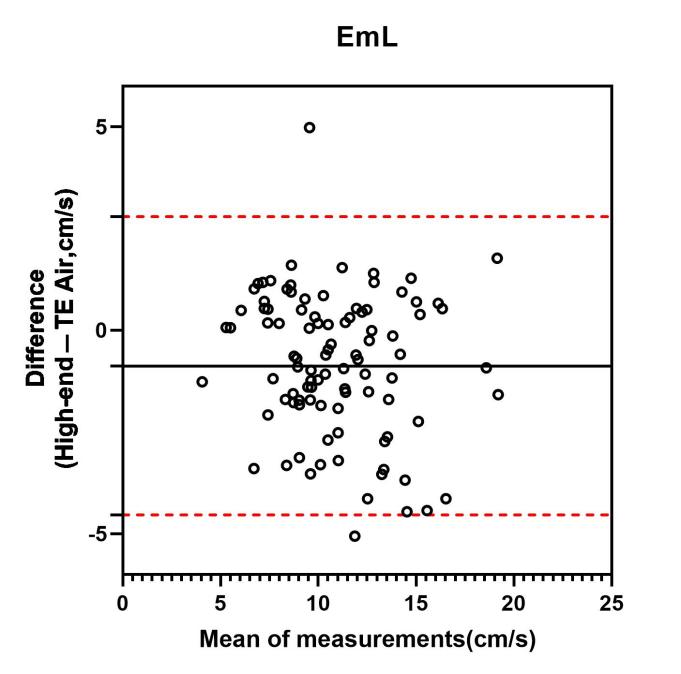 |
| **Supplemental figure 1** Bland-Altman plot of the the High-end device and TE Air device, The y axis represent the difference between the measured values of the two instruments. The x axis represent the average of the measured values of the two device. The solid black line indicates the mean value. Red dashed lines delineate the 95% of cases based on differences between TE Air device and high-end ultrasonic device.AI,Artificial Intelligence; IVSTd,Interventricular septal thickness in diastole; LVPWTd,Left ventricular posterior wall thickness; LVDd,Left ventricular end-diastolic diameter; LVDs,Left ventricular end-systolic diameter; AOD,Aortic diameter; LAD,Left atrial anteroposterior diameter; E and A,Early and late diastolic velocities of the mitral valve; EmS and EmL,Early diastolic velocities at the septal and lateral mitral annulus. | |
